# Possible role of highly activated mucosal NK cells against viral respiratory infections in children undergoing haematopoietic stem cell transplantation

**DOI:** 10.1038/s41598-019-55398-y

**Published:** 2019-12-11

**Authors:** Maria Vela, Teresa del Rosal, Antonio Pérez-Martínez, Jaime Valentín, Inmaculada Casas, Francisco Pozo, Francisco Reinoso-Barbero, David Bueno, Dolores Corral, Ana Méndez-Echevarría, Yasmina Mozo, Cristina Calvo

**Affiliations:** 1 Translational Research in Paediatric Oncology, Hematopoietic Transplantation & Cell Therapy, Hospital La Paz Institute for Health Research (IdiPAZ), Madrid, Spain; 2 Paediatric Infectious Diseases Department, La Paz University Hospital, Madrid, Spain; 3 Paediatric Hemato-Oncology Department, La Paz University Hospital, Madrid, Spain; 4 Respiratory Virus and Influenza Unit, National Microbiology Center (ISCIII), Madrid, Spain; 5 Pediatric Anesthesiology Department, La Paz University Hospital, Madrid, Spain; 6 Translational Research Network in Pediatric Infectious Diseases (RITIP), Madrid, Spain; 7 TEDDY Network (European Network of Excellence for Paediatric Clinical Research), Pavia, Italy

**Keywords:** Viral infection, Paediatric cancer

## Abstract

Infection is the leading cause of non-relapse-related mortality after allogeneic haematopoietic stem cell transplantation (HSCT). Altered functions of immune cells in nasal secretions may influence post HSCT susceptibility to viral respiratory infections. In this prospective study, we determined T and NK cell numbers together with NK activation status in nasopharyngeal aspirates (NPA) in HSCT recipients and healthy controls using multiparametric flow cytometry. We also determined by polymerase chain reaction (PCR) the presence of 16 respiratory viruses. Samples were collected pre-HSCT, at day 0, +10, +20 and +30 after HSCT. Peripheral blood (PB) was also analyzed to determine T and NK cell numbers. A total of 27 pediatric HSCT recipients were enrolled and 16 of them had at least one viral detection (60%). Rhinovirus was the most frequent pathogen (84% of positive NPAs). NPAs of patients contained fewer T and NK cells compared to healthy controls (p = 0.0132 and p = 0.120, respectively). Viral PCR + patients showed higher NK cell number in their NPAs. The activating receptors repertoire expressed by NK cells was also higher in NPA samples, especially NKp44 and NKp46. Our study supports NK cells relevance for the immune defense against respiratory viruses in HSCT recipients.

## Introduction

Allogeneic haematopoietic stem cell transplant (HSCT) is a potentially curative procedure for a variety of pediatric hematologic disorders. Post-transplant infections contribute significantly to the morbidity associated with this procedure. The ablation of HSCT recipient’s immune system by the conditioning regimen increases the susceptibility to infections until they reach immune reconstitution from donor stem cells. The morbidity and mortality from respiratory viruses in immunocompromised patients, including those undergoing HSCT, is well established for respiratory syncytial virus (RSV)^1–4^, parainfluenza viruses (PIV)^5,6^, influenza viruses^7,8^, adenovirus^9^, and human metapneumovirus (HMPV)^4,10^. Recent data suggest that common human coronaviruses (HCoV) are also associated with significant mortality, similar to that seen with RSV, PIV and influenza^11^. There is less information regarding infections caused by human rhinoviruses (HRV) and human bocavirus (HBoV)^12,13^.

Natural Killer (NK) cells in the nasal secretions are a first line of defense against respiratory viruses both in healthy individuals and in those undergoing HSCT. They have a protective role against many viral infections commonly encountered after HSCT, including herpes simplex virus, varicella-zoster virus, cytomegalovirus and influenza virus as well as some bacterial infections^14–16^. NK cells play important roles in the host’s immune defense through the production of cytotoxic granules containing molecules such as perforins and granzymes that lyse infected cells. Other important features of NK cells are the production of cytokines including interferons, their enhancement of local immune responses by directly acting on target cells, and their role in the recruitment and activation of other immune cells, including T cells and macrophages^17^.

NK cells recognize virally infected cells through the integration of signaling from activating and inhibitory germ line encoded receptors on the NK cell surface^17^. Membrane bound Killer-cell Activation Receptors (KARs) work together with inhibitory Killer Immunoglobulin-like Receptors (KIRs), in order to regulate the NK cells functions on infected or transformed cells. The interaction between the KIR family on NK cells and HLA Class I molecules results in inhibitory signals. KARs, like the natural cytotoxicity receptors (NCRs), comprising NKp30, NKp46, and NKp44 are key receptors in the recognition and elimination of virally infected and tumor cells^18,19^.

Because immune cells in the nasal mucosa can control and regulate viral infections via killing infected respiratory epithelial cells, low numbers of NK and or T cells in the nasopharyngeal aspirates (NPAs) of HSCT pediatric recipients could be associated with an increased susceptibility to viral respiratory infections. Although blood immune reconstitution after an HSCT has been widely studied^20–26^, very little is known about the immune reconstitution in HSCT recipients’ mucosa. In the present study, our goals were: 1) to determine NK and T cell numbers in the nasal passages of HSCT recipients; 2) to determine the activation phenotype of NPAs’ NK cells using multiparametric flow cytometry; 3) to determine the presence and functional profile of these cells within the context of a viral infection; and 4) to assess the effects of viral infections on nasal immune reconstitution.

## Materials and Methods

### Study participants

Prospective study including patients under 18 years of age who received allogenic HSCT at La Paz University Hospital (Madrid, Spain), from January 2017 to June 2018. Exclusion criteria included chronic lung disease prior to HSCT. HSCT was performed according to current centre protocols, using bone marrow or peripheral blood stem cells as haematopoietic stem cell source. Acute and chronic graft versus host disease (GVHD) were defined and graded as previously reported^27,28^.

Age- and sex-matched controls were recruited among healthy patients undergoing elective surgery. Cases and controls were also matched according to procedure date (HSCT and elective surgery, respectively) ± 1 week.

All research was performed in accordance to local regulations. Informed consent in accordance with the Declaration of Helsinki was obtained from the parents of all children. The study was approved by the Clinical Research Ethics Committee at La Paz University Hospital.

### Study samples

Study samples were peripheral blood (PB) and NPAs that were collected prior to the conditioning regimen and after HSCT at days 15 and 30 for PB mononuclear cells and before the conditioning regimen and at days 0, 10, 20 and 30 after HSCT for NPAs. The following tests were performed in each sample type:

- PB: total, T and NK cell counts.
- NPAs: total T and NK cell counts, natural cytotoxicity receptors, viral detection.

For NPA collection, 1 mL of 0.9% saline solution was instilled into each nostril and the nasopharynx was suctioned using a sterile catheter with a mucus trap. NPAs were not performed if the patient had active bleeding or his platelet count was below 20.000/mm3. NPA samples containing blood were considered not valid and discarded. Each specimen was sent to the Respiratory Virus and Influenza Unit at the National Microbiology Center (ISCIII, Madrid, Spain) for viral analysis and to the Translational Research in Paediatric Oncology, Hematopoietic Transplantation & Cell Therapy Unit at La Paz Hospital Research Institute (Madrid, Spain) for flow cytometry assays. NPAs were processed within 24 hours after collection. In the control group, NPAs were collected on the day of surgery.

### Viral detection by RT-PCR

Upon reception of the samples, three aliquots were prepared and stored at −80 °C. Both the reception and the NPA sample processing areas are separated from those defined as working areas. Three independent RT-PCR assays were performed to detect sixteen respiratory viruses as previously published by our group^29–31^. Influenza A, B and C viruses were detected by using previously described primer sets only to amplify influenza viruses in a multiplex PCR assay^29^. A second multiplex PCR was used to detect parainfluenza viruses 1 to 4, human coronaviruses 229E and OC43, enteroviruses and HRV^30^. Presence of RSV A and B types, HMPV, HBoV and adenoviruses was established using a third multiplex RT-nested PCR−BRQ method^31^.

### Cell characterization by flow cytometry

For flow cytometry assays, NPA samples were filtered with a 70 µm cell strainer (Falcon), centrifuged at 1400 rpm (5 min) and suspended in 100 µl of PBS. Total sample volume was acquired using a Navios flow cytometer (Beckman Coulter).

The total cell count per sample was determined. T cell (CD45+, CD3+ CD56−) and Natural killer (NK) cell (CD45+, CD56+, CD3−) populations were identified using the following labelled antibodies: CD45-FITC/APCCy7 (clone 2D1, BD Pharmingen), CD56-APC (clone NCAM16.2, BD Pharmingen) and CD3-PECy7 (clone UCHT1, Biolegend). Fluorescent cell viability dye 7-AAD (BD Pharmingen) was used to exclude dead cells. FlowJo v10.0.7 software (TreeStar) was used for data analysis.

The expression of natural cytotoxicity receptors (NCRs), NKG2A and NKG2D receptors on NK cells subpopulation in NPA was evaluated with the following fluorochrome-conjugated antibodies: NKG2D CD314-PE (clone BAT221, MiltenyiBiotec); NKp46 CD335-FITC (clone 9E2, AbDSerotec); NKp44 CD-336-PE (clone 44, BD Pharmingen); NKp30 CD337-PE (clone REA823, MiltenyiBiotec); NKG2A-PE (clone 131411, R&D systems; T1, Biolegend).

### Statistical analysis

Values are expressed as percentages for discrete variables and as median and interquartile range (IQR) for continuous variables. Comparisons between groups (controls and HSCT patients before conditioning, and patients with and without viral respiratory infections) were made using Mann-Whitney U test, and Fisher’s exact test) as appropriate. A value of *P* < 0.05 was considered statistically significant. All analyses were performed using the SPSS statistical software, version 21.0 (IBM Corp.).

## Results

### HSCT recipients and control group characteristics

During the study period, 27 HSCT recipients were recruited. Their main characteristics are summarized in Table 1. The median age of patients was 7.7 years (IQR 9.2).

**Table 1:** Characteristics of haematopoietic stem cell transplantation recipients (n = 27).

Age (years)	6 (9.8)
Female gender	14 (52)
**Indication for HSCT**	
PID	9 (33)
SAA	5 (19)
ALL	5 (19)
AML	2 (7)
MDS	3 (11)
Other	3 (11)
**Donor type**	
MRD	4 (15)
MMRD	8 (30)
MUD	12 (44)
MMUD	3 (11)
**Graft source**	
Bone marrow	14 (52)
Mobilized peripheral blood	13 (48)
**Conditioning regimen**	
Reduced intensity	17 (63)
Myeloablative	10 (37)
GVHD prophylaxis*	25 (93)
*In vivo:* ATG/alemtuzumab	14 (52)
*Ex vivo:* T-celldepletion (CD45RA/ TCRαβ)	11 (41)

Data are n (%) or median (interquartile range) where appropriate.

Abbreviations: HSCT, haematopoietic stem cell transplantation**;** PID, primary immunodeficiency; SAA, severe aplastic anemia; ALL, acute lymphoblastic leukaemia; AML, acute myeloid leukaemia; MDS, myelodysplastic syndrome; MRD, matched related donor; MMRD, mismatched related donor; MUD, matched unrelated donor; MMUD, mismatched unrelated donor; GVHD, graft versus host disease; ATG, anti-thymocyte globulin.

*Two patients transplanted from HLA-identical siblings received neither ATG nor T-cell depletion.

All HSCT were allogenic. Twenty-five patients underwent HSCT for the first time (93%) and two received their second HSCT (7%).

Eighteen healthy children were included in the control group, 10 male (56%) and 8 female (44%), with a median age of 8.7 years (IQR 9). There were no significant differences regarding age and sex between HSCT recipients and healthy controls.

### Viral infections

A total of 83 samples were collected from the 27 HSCT recipients, and 77 were valid for viral studies (median number of valid samples per patient: 3; IQR 2). Twenty-five samples (32%) were positive, and 16 of 27 HSCT recipients (60%) had at least one viral detection. Among HSCT recipients with viral infection, the median number of positive samples per patient was 1 (IQR 1). HRV was isolated in 21 samples (84% of positive NPA) from 12 patients, followed by adenovirus and parainfluenza type 1 (two positive samples from two different patients each, 8%). There were no viral coinfections among HSCT recipients. Detailed information regarding positive samples is given in Table 2.

**Table 2:** Samples with positive viral detection.

Sample type		Valid samples*	Positive samples (%)	Respiratory viruses (n°)
HSCT recipients	Day 7	20	9 (45)	HRV (6), ADV (2), PIV (1)
	Day 0	21	6 (29)	HRV (6)
	Day 10	15	3 (20)	HRV (3)
	Day 20	12	4 (33)	HRV (4)
	Day 30	6	3 (50)	HRV (2), PIV (1)
	After day 30	3	0	
Healthy controls		17	4 (24)	HRV (1), ADV (1), HRV + AV (1), HRV + HBoV (1)

Abbreviations: ADV, adenovirus; HBoV, human bocavirus; HRV, human rhinovirus; PIV, parainfluenza virus.

*A total of five samples were not valid because they contained blood or because polymerase chain reaction was inhibited.

Infections caused by HRV were symptomatic in 2 of 12 patients (17%): one had low-grade fever and the other persistent rhinorrhea. Both patients with adenovirus infections had fever, mucositis and elevated levels of C-reactive protein (above 100 mg/L). Infections by parainfluenza type 1 virus were also symptomatic (one patient with fever and another with laryngitis and pneumonia). None of the patients required admission to the intensive care unit (ICU) nor died as a result of a viral infection. There were no differences regarding age between HSCT recipients with and without viral infections (median [IQR] 7.5 [8.8] and 6 [10.2] years of age, respectively, p = 0.94), but patients below two years of age tested positive more frequently (11/21 samples, 52% vs. 14/56, 25%, p = 0.03).

A total of 17 samples from healthy controls were analyzed, and viruses were identified in 4 (24%): two single infections (HRV and adenovirus) and two coinfections (HRV and HBoV, HRV and adenovirus) (Table 2). Controls with viral infections were younger, but this difference did not reach statistical significance (median [IQR] 4.1 [6.6] vs. 8.9 [8.5] years, p = 0.07). All infections were asymptomatic.

No significant differences were found regarding viral isolation rate between patients and healthy controls (32% and 24% of samples, respectively, p = 0.57).

### NPA cellular composition of HSCT recipients

**Figure 1:**
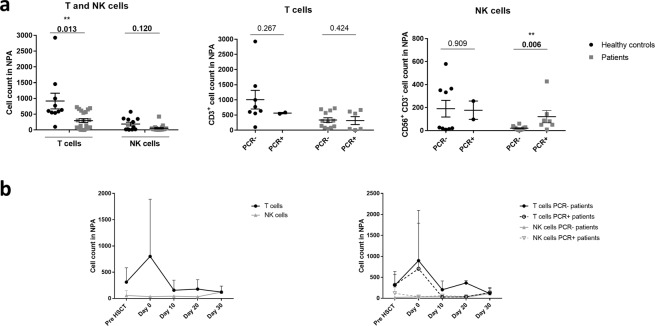
T and NK cells in nasopharyngeal aspirates (NPAs) of patients prior to the HSCT conditioning are reduced as compared to healthy controls and have similar post HSCT kinetics in patients with and without viral respiratory infection. (**a**) Total number of T (CD45+, CD3+ CD56−) and NK cells (CD45+, CD3-, CD56+) in NPA of healthy controls and patients prior to the HSCT conditioning was determined by multiparametric flow cytometry. (**b**) Number of T and NK cells in NPAs was also monitored for one month post HSCT.

The NPAs of patients prior to HSCT conditioning contained fewer T and NK cells as compared with healthy controls p = 0.0132 and p = 0.120, respectively (Fig. 1A). Additionally, PCR + patients prior to HSCT conditioning showed statistically significant higher numbers of NK cells in NPAs than PCR− patients (p = 0.006). Those differences were not observed in healthy controls or in the T cell populations isolated from NPAs.

We further analyzed T and NK cell kinetics after the HSCT. We observed an increase in T cells in the NPA samples at infusion day. This increase is reverted at day 10 post HSCT, when NPA cell counts reach levels similar to those observed prior to the HSCT. This effect was observed both in PCR+ and in PCR− patients (Fig. 1B and Suppl. Table 1). However, we did not observe that peak with NK cells. There were no differences regarding NPA T and NK cell counts over time in patients with and without respiratory infection.

### PB cellular composition of HSCT recipients

**Figure 2:**
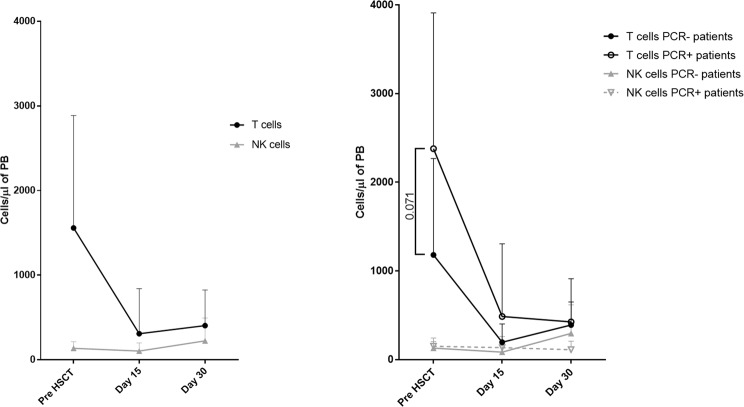
T and NK cells in peripheral blood (PB) of HSCT recipients. Number of T (CD45+, CD3+ CD56-) and NK cells (CD45+, CD3−, CD56+) in PB of HSCT patients was determined by multiparametric flow cytometry. Differences in T and NK cells numbers in PB of patients with PCR− and PCR+ for respiratory virus are indicated (n = 18).

Prior to the HSCT, the initial T cell counts were higher in PCR+ patients than in PCR− patients (median, IQR 2014, 3677-1090 vs. 945, 1751-249 cells/µl, p = 0.07)) (Fig. 2 and Suppl. Table 2). However, PCR + and PCR− patients had similar initial NK cells counts prior to the HCT (median, IQR 154, 225-61 vs. 111, 185-62 cells/µl). Median T cell counts decreased notably after the HSCT, both in PCR+ and PCR− patients and on day 30 post HSCT the numbers were still far from the initial values. This effect was not observed in PB NK cell counts either.

### Natural cytotoxicity receptors (NCRs) expression in NPA’s NK cells

**Figure 3:**
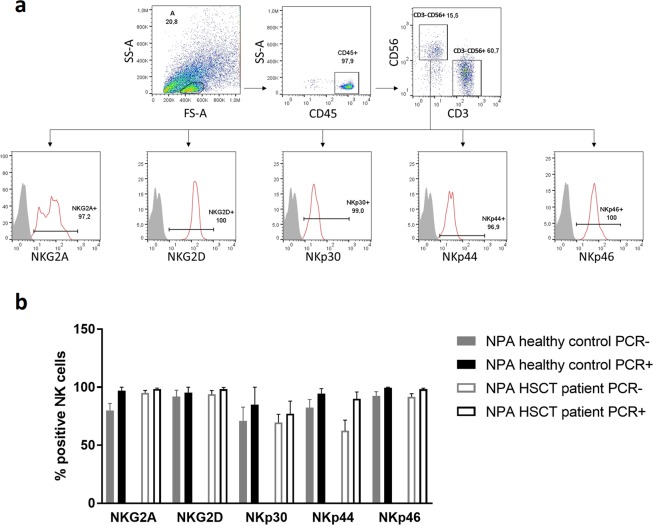
NK cells in NPAs have a high activating status especially in PCR+ samples. (**a**) One representative flow cytometry analysis of an HSCT recipient NPA. CD45+, CD3−, CD56− cells were analyzed for the indicated NCRs (red lines) and compared with fluorescence minus one controls (grey). **(b)** Percentage of NK cells expressing activating receptors in PCR+ and PCR− NPAs of healthy controls versus patients prior to the HSCT.

The NK cells found in the NPA samples had a highly activated status as determined by flow cytometry analysis (Fig. 3A). The percentage of NK cells expressing different activating receptors was higher in PCR + NPA samples than in PCR− samples for all the receptors analyzed, and especially significant for NKp44 and NKp46 receptors (Fig. 3B). The highly activated status was observed both in healthy donors and HSCT patients’ NPAs.

### HSCT recipient outcomes

The outcomes of HSCT recipients are summarized in Table 3. There were no significant differences according to the presence of viral infection. The number of T or NK cells in patients’ NPAs pre-, during- or after-HSCT was not correlated with a higher risk of GVHD, graft failure, ICU admission or death (Suppl. Tables 3 and 4).

**Table 3:** Outcomes of haematopoietic stem cell transplantation recipients.

	All cases	Positive viral detection	Negative viral detection	p
**Number of cases**	27	16	11	
**Acute GVHD**	11 (41)	7 (44)	4 (36)	1
I-II	4 (15)			
III-IV	7 (26)			
**Chronic GVHD**	3 (11)	2 (13)	1 (9)	1
Mild	1 (4)			
Moderate	1 (4)			
Severe	1 (4)			
Graft failure	2 (7)	1 (6)	1 (9)	1
ICU admission	7 (26)	2 (12)	5 (45)	0.08
Death	7 (26)	3 (19)	4 (36)	0.391

Data are n (%).

Abbreviations: GVHD, graft versus host disease; ICU, intensive care unit.

## Discussion

The nasal mucosa is the first place within the respiratory system to be exposed to inhaled viral pathogens. Therefore, nasal immune cells are likely to play an important role in early innate immune responses to those environmental factors, both in healthy individuals and in immunocompromised patients like HSCT recipients. NK cells are thought to play a particularly important role in this context, since they are able to influence both innate and adaptive immune responses^32,33^ and are the first lymphocyte subset to appear in PB after HSCT^20–26^. While macrophages and dendritic cells have been identified in the nasal submucosa^34^, and neutrophils have been identified in the nasal cavity^35^, the immune cells present in NPAs have not been fully characterized. To phenotype NPA cells, previous researchers have relied on cell differential analysis of cytocentrifuge slides stained with haematoxylin and eosin. However, flow cytometry may be a superior method to fully characterize NPA immune cells since it allows for a precise quantification of cell populations as well as for phenotype studies, making it possible to determine the activation status of such specific cell populations.

Our study showed that HSCT patients, as expected -due to conditioning regimens and underlying disease- had fewer T and NK cells in their NPAs as compared to healthy controls. Interestingly, those patients exposed to viral pathogens showed statistically significant higher NK cell counts in their NPAs, which is in accordance with their role in the “front line” defense against viral infections. The phenotypes and functions of NK cells differ depending on the source organ or tissue^36–38^. Our current research further explores the particular phenotype of nasal NK cells, which show a higher activation status than those found in PB, comparing the results obtained in this study with previous publications from our group^24^. This was especially true for those individuals with positive viral detection in their nasal secretions. Okada *et al*. recently reported that NK cells found in the nasal passages of mice belong to the conventional NK cell linage and characteristically demonstrate an immature and activated phenotype^39^. Our results constitute the first evidence of such observations in humans.

In our study, we observed an unexpected T cells increase in NPAs on HSCT day 0 samples, despite conditioning regimens. We attribute it to the fact that all NPA samples were collected in the morning, and almost all patients receiving non-manipulated grafts (60% of our cohort) had finished their infusions prior to NPA collection. Pre-transplant conditioning, such as high-dose chemotherapy is toxic to many cells and render the endothelium vulnerable^40^ which could lead to increased T cells in NPA of many patients.

Natural killer cell activity is regulated through the balance of signals from inhibitory and activating receptors. The natural cytotoxicity receptors (NCRs) NKp46 (NCR1), NKp44 (NCR2), and NKp30 (NCR3), as well as NKG2D, are the main activating receptors involved in mediating NK cell function in health and disease^18,19^. The most overexpressed activating receptors we found in NK cells of NPAs were NKp44 (NCR2) and NKp46 (NCR1). This is consistent with Shemer-Avni *et al*.’s findings^41^. In nasal lavage samples of patients infected with respiratory viruses, they found NKp46 as the most abundantly expressed NCR. However, in their RTqPCR analysis they detected no NKp44 expression and very little of NKp30. Altogether, our findings point to NKp44 as a key player in nasal mucosa elimination of virally infected cells by NK cells.

Conditioning regimens used for allogeneic HSCT, high dose chemotherapy and/or radiotherapy, induce serious damage in mucosal, humoral and cellular immune function. Moreover, those regimens can harm the host’s thymopoiesis resulting in further delayed immune recovery^25,26^. Collectively, these factors predispose the host to a variety of infections. In fact, despite the routine use of prophylactic antimicrobials in the peri-transplant period^42,43^, infections occur is about 80–85% of HSCT recipients and are one of the leading causes of non-relapse-related mortality after allogeneic HSCT^44,45^.

More than half of the HSCT recipients included in our study presented a viral respiratory infection at some point. To date, most studies of these infections in pediatric HSCT recipients have focused on symptomatic individuals, reporting an incidence of 5–11%^46–48^. Asymptomatic respiratory infections in children undergoing HSCT have been barely studied. A prospective study of 33 patients detected respiratory viruses in 24% of them before HSCT, mainly HRV and adenovirus^49^. In our series, infections caused by HRV were the most common, and were not associated with increased morbidity. These findings are in concordance with those reported by Srinivasan *et al*. in children with cancer or post-HSCT and upper or lower respiratory infection^13^ and asymptomatic children before HSCT^49^. Other authors have reported implementing routine extensive pre-HSCT lung screening, including bronchoalveolar lavage for the detection of viruses, bacteria and fungi. In that study, almost one third of asymptomatic patients had viral respiratory infections, mainly caused by rhinovirus, and none of these worsened during HSCT^50^. On the other hand, infections caused by adenovirus and parainfluenza virus were less frequent but more often symptomatic in our patients. Adenovirus has been described as a cause of lower respiratory tract infection with high mortality after HSCT in studies involving symptomatic children^46,51^. We did not find a correlation between viral infection and HSCT outcomes, which could be caused by small sample size and low incidence of symptomatic infection.

Important strengths of our study include its prospective design and the determination of number, activation phenotype and functional profile of NK cells together with respiratory virus detection, assessing HSCT recipients over time and comparing them with healthy controls. Potential limitations of our study relate to sample size and losses to follow-up. NPA cell counts have great variability so larger cohorts would be desirable to draw stronger conclusions. Many patients did not tolerate well NPAs and withdrew during the course of the study. The small number of patients further precluded correlation of viral detection and transplantation outcomes, and limits our ability to examine the implications of asymptomatic viral respiratory infections in HSCT recipients. NPAs were collected up to 30 days after HSCT, and some infections occurring later may have been missed. However, most respiratory viral infections in HSCT recipients occur soon after transplantation, and adverse clinical outcomes are more likely to occur in the same period^46,51^.

In conclusion, we provide data suggesting NK cells as major effectors in respiratory viral control in the nasal mucosa. We also characterized this NK cell population finding a highly activated phenotype compared with that of their PB counterparts. Further study of this unique mucosal immune cell population will be beneficial in assessing the effects of viral infections on upper respiratory immune responses in HSCT recipients as well as in healthy individuals.

## Supplementary information

Supplementary material
