# Supplementary material for: Possible role of highly activated mucosal NK cells against viral respiratory infections in children undergoing haematopoietic stem cell transplantation
Source: Sci Rep. 2019 Dec 11;9:18792. doi: 10.1038/s41598-019-55398-y (PMC6906525; doi:10.1038/s41598-019-55398-y)
Supplement: Supplementary file 1 — Supplementary material [file 41598_2019_55398_MOESM1_ESM.pdf]

**TITLE**

Possible role of highly activated mucosal NK cells against viral respiratory infections in children undergoing haematopoietic stem cell transplantation

**AUTHORS**

Maria Vela[*1], Teresa del Rosal[*2], Antonio Pérez-Martínez[#3], Jaime Valentín[4], Inmaculada Casas[5], Francisco Pozo[6], Francisco Reinoso-Barbero[7], David Bueno[8], Dolores Corral[9], Ana Méndez-Echevarría[10], Yasmina Mozo[11], Cristina Calvo[12].

1. Maria Vela[*] PhD; Translational Research in Paediatric Oncology, Hematopoietic Transplantation & Cell Therapy, Hospital La Paz Institute for Health Research (IdiPAZ), Madrid, Spain. maria.vela@idipaz.es
2. Teresa del Rosal[*] MD, PhD; Paediatric Infectious Diseases Department, La Paz University Hospital, Madrid, Spain; Hospital La Paz Institute for Health Research (IdiPAZ), Madrid, Spain. teredelrosal@yahoo.es
3. Antonio Pérez-Martínez[#] MD, PhD; Paediatric Hemato-Oncology Department, La Paz University Hospital, Madrid, Spain; Translational Research in Paediatric Oncology, Hematopoietic Transplantation & Cell Therapy, Hospital La Paz Institute for Health Research (IdiPAZ), Madrid, Spain. aperezmartinez@salud.madrid.org
4. Jaime Valentín MSc; Translational Research in Paediatric Oncology, Hematopoietic Transplantation & Cell Therapy, Hospital La Paz Institute for Health Research (IdiPAZ), Madrid, Spain. jaimevquiroga@hotmail.com
5. Inmaculada Casas PhD. Respiratory Virus and Influenza Unit, National Microbiology Center (ISCIII), Madrid, Spain. icasas@isciii.es
6. Francisco Pozo PhD. Respiratory Virus and Influenza Unit, National Microbiology Center (ISCIII), Madrid, Spain. pacopozo@iscii.es

7. Francisco Reinoso-Barbero MD, PhD; Pediatric Anesthesiology Department, La Paz University Hospital, Madrid, Spain. francisco.reinoso@salud.madrid.org
8. David Bueno MD; Paediatric Hemato-Oncology Department, La Paz University Hospital, Madrid, Spain. david.bueno@salud.madrid.org
9. Dolores Corral MD, PhD; Paediatric Hemato-Oncology Department, La Paz University Hospital, Madrid, Spain. mariadolores.corral@salud.madrid.org
10. Ana Méndez-Echevarría MD, PhD; Paediatric Infectious Diseases Department, La Paz University Hospital, Madrid, Spain; Hospital La Paz Institute for Health Research (IdiPAZ), Madrid, Spain. amendezes@yahoo.es
11. Yasmina Mozo MD; Paediatric Hemato-Oncology Department, La Paz University Hospital, Madrid, Spain. yasmina.mozo@salud.madrid.org
12. Cristina Calvo MD, PhD; Paediatric Infectious Diseases Department, La Paz University Hospital, Madrid, Spain; Hospital La Paz Institute for Health Research (IdiPAZ), Madrid, Spain. Translational Research Network in Pediatric Infectious Diseases (RITIP), Madrid, Spain. TEDDY Network (European Network of Excellence for Paediatric Clinical Research), Italy. ccalvorey@gmail.com

* These authors contributed equally to this work.

# Corresponding Author Address:

Antonio Pérez-Martínez MD, PhD. Paediatric Hemato-Oncology Department. La Paz University Hospital. Paseo de la Castellana, 261, Madrid, 28046, Spain. Phone: +34917277223; fax: +34917277042. Email: aperezmartinez@salud.madrid.org

**Supplementary Table 1. T cell and NK cell counts in patients' NPAs.**

| | pre-HSCT (total cell counts) | Day 0 (total cell counts) | Day 10 (total cell counts) | Day 20 (total cell counts) | Day 30 (total cell counts) |
|---|---|---|---|---|---|
| T | 294, 614-45 | 361, 1827-6 | 56, 296-25 | 56, 366-35 | 65, 242-32 |
| NK | 26, 60-9 | 38, 56-20 | 46, 69-20 | 26, 51-16 | 13, 84-11 |

| | pre-HSCT (total cell counts) | Day 0 (total cell counts) | Day 10 (total cell counts) | Day 20 (total cell counts) | Day 30 (total cell counts) |
|---|---|---|---|---|---|
| T_PCR- | 294, 610-82 | 532, 1827-338 | 116, 362-49 | 366, 423-309 | 59, 279-5 |
| T_PCR+ | 294, 626-32 | 7, 2103-4 | 25, 52-17 | 38, 52-22 | 135, 206-65 |
| p value | 0.624 | 0.343 | 0.133 | 0.057 | 0.800 |

| | pre-HSCT (total cell counts) | Day 0 (total cell counts) | Day 10 (total cell counts) | Day 20 (total cell counts) | Day 30 (total cell counts) |
|---|---|---|---|---|---|
| NK_PCR- | 18, 27-6 | 44, 56-11 | 40, 58-15 | 21, 44-14 | 13, 79-10 |
| NK_PCR+ | 80, 143-50 | 34, 60-22 | 69, 69-47 | 51, 51-26 | 51, 90-12 |
| p value | **0.005** | 0.960 | 0.164 | 0.143 | 0.800 |

Data are median, IQR.

Abbreviations: NK_PCR-, NK cell count in HSCT recipients without viral respiratory infection; NK_PCR+, NK cell count in HSCT recipients with viral respiratory infection; T_PCR-, T cell count in HSCT recipients without viral respiratory infection; T_PCR+, T cell count in HSCT recipients with viral respiratory infection.

**Supplementary Table 2. T cell and NK cell counts in patients' PB.**

| | pre-HSCT (cells/µl) | Day 15 (cells/µl) | Day 30 (cells/µl) |
|---|---|---|---|
| T | 1127, 2263-591 | 113, 415-6 | 306, 538-143 |
| NK | 128, 192-68 | 66, 194-23 | 122, 325-65 |

| | pre-HSCT (cells/µl) | Day 15 (cells/µl) | Day 30 (cells/µl) |
|---|---|---|---|
| T_PCR- | 954, 1751-249 | 129, 391-7 | 238, 408-93 |
| T_PCR+ | 2014, 3677-1090 | 63, 846-3 | 448, 596-314 |
| p value | 0.071 | 0.929 | 0.210 |

| | pre-HSCT (cells/µl) | Day 15 (cells/µl) | Day 30 (cells/µl) |
|---|---|---|---|
| NK_PCR- | 111, 185-62 | 63, 140-17 | 168, 348-99 |
| NK_PCR+ | 154, 225-61 | 78, 272-25 | 82, 111-56 |
| p value | 0.616 | 0.374 | 0.085 |

Data are median, IQR.

Abbreviations: NK_PCR-, NK cell count in HSCT recipients without viral respiratory infection; NK_PCR+, NK cell count in HSCT recipients with viral respiratory infection; T_PCR-, T cell count in HSCT recipients without viral respiratory infection; T_PCR+, T cell count in HSCT recipients with viral respiratory infection.

**Supplementary Table 3. NPA T cell reconstitution versus outcome.**

| | **pre-HSCT** (total cell counts) | **Day 0** (total cell counts) | **Day 10** (total cell counts) | **Day 20** (total cell counts) | **Day 30** (total cell counts) |
|---|---|---|---|---|---|
| **No acute GVHD** | 86, 580-7 (12) | 11, 1046-4 (8) | 36, 53-15 (8) | 211, 534-50 (4) | 65 (1) |
| **Acute GVHD** | 294, 309-110 (7) | 384, 396-349 (3) | 80, 116-52 (3) | 18, 30-9 (3) | 5 (1) |
| p value | 0.605 | 0.630 | 0.303 | 0.114 | - |
| | | | | | |
| **No Chronic GVHD** | 141, 547-41 (17) | 164, 402-6 (10) | 36, 73-19 (10) | 38, 52-22 (6) | 35, 50-20 (2) |
| **Chronic GVHD** | 348, 293-238 (2) | 2800.0 (1) | 56.0 (1) | 1038.0 (1) | - |
| p value | 0.632 | - | - | - | - |
| | | | | | |
| **No graft failure** | 134, 489-41 (18) | 13, 384-5 (9) | 36, 55-19 (10) | 38, 285-22 (6) | 35, 50-20 (2) |
| **Graft failure** | 690 (1) | 1436, 1826-1046 (2) | 296 (1) | 56 (1) | - |
| p value | - | 0.146 | - | - | - |
| | | | | | |
| **No ICU admission** | 80, 431-24 (15) | 10, 164-4 (7) | 47, 56-17 (9) | 42, 366-35 (5) | 35, 50-20 (2) |
| **ICU admission** | 492, 698-255 (4) | 532, 1046-402 (4) | 160, 228-92 (2) | 28, 42-14 (2) | - |
| p value | 0.077 | 0.073 | 0.455 | 0.571 | - |
| | | | | | |
| **Alive** | 62, 489-15 (14) | 10, 164-4 (7) | 36, 53-16 (8) | 42, 366-35 (5) | 35, 50-20 (2) |
| **Dead** | 303, 690-294 (5) | 532, 1046-402 (4) | 80, 188-52 (3) | 28, 42-14 (2) | - |
| p value | 0.066 | 0.073 | 0.206 | 0.571 | - |

Data are summarized as median, IQR (n).

Abbreviations: HSCT: haematopoietic stem cell transplantation; GVHD, graft versus host disease; ICU, intensive care unit.

**Supplementary Table 4. NPA NK cell reconstitution versus outcome.**

| | **pre-HCT** (total cell counts) | **Day 0** (total cell counts) | **Day 10** (total cell counts) | **Day 20** (total cell counts) | **Day 30** (total cell counts) |
|---|---|---|---|---|---|
| **No acute GVHD** | 44, 81-14 (12) | 30, 44-20 (6) | 43, 52-16 (8) | 25, 32-25 (4) | 12.0 (1) |
| **Acute GVHD** | 20, 38-8 (7) | 44, 53-37 (3) | 57, 63-51 (3) | 16, 33-12 (3) | 79.0 (1) |
| p value | 0.253 | 0.417 | 0.511 | 0.714 | - |
| | | | | | |
| **No Chronic GVHD** | 28, 60-11 (17) | 38, 30-30 (10) | 47, 69-40 (10) | 25, 44-18 (6) | 45, 62-28 (2) |
| **Chronic GVHD** | 78, 115-41 (2) | 20.0 (1) | 13.0 (1) | 14.0 (1) | - |
| p value | 0.983 | - | - | - | - |
| | | | | | |
| **No graft failure** | 32, 75-9 (18) | 41, 53-30 (10) | 47, 69-40 (9) | 20, 25-14 (6) | 45, 62-28 (2) |
| **Graft failure** | 28 (1) | 11, 15-6 (2) | 10 (1) | 51 (1) | - |
| p value | - | 0.067 | - | - | - |
| | | | | | |
| **No ICU admission** | 39, 83-9 (15) | 44, 59-32 (7) | 47, 69-40 (9) | 25, 26-14 (5) | 45, 62-28 (2) |
| **ICU admission** | 24, 36-17 (4) | 25, 33-15 (4) | 10.0 (1) | 33, 42-24 (2) | - |
| p value | 0.866 | 0.152 | - | 0.619 | - |
| | | | | | |
| **Alive** | 44, 85-10 (14) | 44, 59-32 (7) | 47, 69-34 (8) | 25, 26-14 (5) | 45, 62-28 (2) |
| **Dead** | 20, 28-11 (5) | 25, 33-15 (4) | 28, 37-19 (3) | 33, 42-24 (2) | - |
| p value | 0.331 | 0.152 | 0.200 | 0.619 | - |

Data are summarized as median, IQR (n).

Abbreviations: HSCT: haematopoietic stem cell transplantation; GVHD, graft versus host disease; ICU, intensive care unit.
